# Increased Pleiotrophin Concentrations in Papillary Thyroid Cancer

**DOI:** 10.1371/journal.pone.0149383

**Published:** 2016-02-25

**Authors:** Youn Hee Jee, Samira M. Sadowski, Francesco S. Celi, Liqiang Xi, Mark Raffeld, David B. Sacks, Alan T. Remaley, Anton Wellstein, Electron Kebebew, Jeffrey Baron

**Affiliations:** 1 *Eunice Kennedy Shriver* National Institute of Child Health and Human Development, National Institutes of Health, Bethesda, Maryland, United States of America; 2 Endocrine Oncology Branch, National Cancer Institute, National Institutes of Health, Bethesda, Maryland, United States of America; 3 Division of Endocrinology and Metabolism, Virginia Commonwealth University, Richmond, Virginia, United States of America; 4 Center for Cancer Research, National Cancer Institute, National Institutes of Health, Bethesda, Maryland, United States of America; 5 Department of Laboratory Medicine, Clinical Center, National Institutes of Health, Bethesda, Maryland, United States of America; 6 Department of Oncology, Georgetown University Medical Center and Lombardi Comprehensive Cancer Center, Washington, District of Columbia, United States of America; University of Connecticut Health Center, UNITED STATES

## Abstract

**Background:**

Thyroid nodules are common, and approximately 5% of these nodules are malignant. Pleiotrophin (PTN) is a heparin-binding growth factor which is overexpressed in many cancers. The expression of PTN in papillary thyroid cancer (PTC) is unknown.

**Method and Findings:**

74 subjects (age 47 ± 12 y, 15 males) who had thyroidectomy with a histological diagnosis: 79 benign nodules and 23 PTCs (10 classic, 6 tall cell, 6 follicular variant and 1 undetermined). Fine-needle aspiration (FNA) samples were obtained *ex vivo* from surgically excised tissue and assayed for PTN and thyroglobulin (Tg). Immunohistochemistry (IHC) was performed on tissue sections. In FNA samples, PTN concentration normalized to Tg was significantly higher in PTC than in benign nodules (16 ± 6 vs 0.3 ± 0.1 ng/mg, p < 0.001). In follicular variant of PTC (n = 6), the PTN/Tg ratio was also higher than in benign nodules (1.3 ± 0.6 vs 0.3 ± 0.1 ng/mg, *P* < 0.001, respectively). IHC showed cytoplasmic localization of PTN in PTC cells.

**Conclusion:**

In *ex vivo* FNA samples, the PTN to thyroglobulin ratio was higher in PTCs, including follicular variant PTC, than in benign thyroid nodules. The findings raise the possibility that measurement of the PTN to Tg ratio may provide useful diagnostic and/or prognostic information in the evaluation of thyroid nodules.

## Introduction

Thyroid nodules occur frequently in the general population with a prevalence of approximately 3–7% for palpable masses [1, 2]. Approximately 5% of thyroid nodules are malignant [3] and the most common histological type is papillary thyroid cancer (PTC) [1]. Two major challenges facing clinicians are to distinguish malignant from benign nodules and to identify those thyroid malignancies that are aggressive [1]. Fine needle aspiration (FNA) cytology represents the primary preoperative diagnostic tool for the evaluation of thyroid nodules [4], but it is inconclusive in up to 30% of patients [5]. In particular, follicular variant PTC is difficult to distinguish from benign follicular lesions by cytology [6, 7].

Pleiotrophin (PTN) and midkine (MDK) are related polypeptide heparin-binding growth factors [8, 9]. PTN and MDK are overexpressed in various human cancers, where they are thought to promote cell survival, proliferation and angiogenesis, contributing to tumor growth [10, 11].

We recently reported that the concentration of MDK in FNA samples is elevated in PTCs compared to benign nodules [12]. In that study, the MDK concentration was normalized to the thyroglobulin (Tg) concentration, which adjusted for tissue content and also enhanced the separation between malignant and benign samples because of lower Tg concentrations in malignant nodules. However, neither the MDK concentration nor the MDK/Tg ratio was elevated in the follicular variant of PTC subgroup [12], limiting the potential diagnostic value of this approach.

PTN was previously reported to be overexpressed in medullary thyroid cancer [13], but the expression of PTN in PTCs has not been investigated. We hypothesized that PTN concentration and PTN/Tg concentration ratio are higher in PTCs than in benign nodules.

## Materials and Methods

### Subjects and sample collection

Seventy-four adult subjects (age 47 ± 12 y, 15 males) with thyroid nodules who underwent thyroidectomy at the National Institutes of Health (NIH) Clinical Center were included in the analysis. Study protocols were approved by National Institute of Diabetes and Digestive and Kidney Disease Institutional Review Board, and all patients provided written informed consent to participate in the study. After the thyroid was excised, selected nodules with surrounding tissues were bisected for procurement and then *ex vivo* FNA was performed by passing a 25-gauge needle into the nodules. The needle was passed 10 to 20 times. No suction was applied. The tissue within the needle was washed out with 0.5 ml of PBS containing 1% BSA. The samples were aliquoted and stored immediately at –80 C until assay. Multiple *ex vivo* FNA samples (mean, 3.0 samples) were obtained per nodule. We initially attempted to measure PTN in *in vivo* FNA washout samples, after the needle contents had been expelled for cytology, but often found undetectable PTN concentrations, indicating insufficient tissue remaining in the needle (data not shown).

A total of 103 nodules were sampled. Of these, 62 nodule samples were previously assayed for MDK concentration and included in a prior report [12].

### Pleiotrophin Sandwich ELISA Assay

A PTN sandwich ELISA was developed in our laboratory (see S1 Method for assay details). The intra-assay CV was 6.9% for high concentration (1.3 ng/mL) and 9% for low concentration (0.2 ng/mL). The inter-assay CV was 8.8% at 0.2 ng/mL and 12.3% at 0.6 ng/mL. The limit of detection was 10 pg/mL. There was no cross-reactivity with up to 50 ng/mL of MDK (S1A Fig). PTN concentrations in PBS containing 1% BSA remained stable in plastic but not glass tubes over 2 hours at room temperature and with repeated freeze-thaw cycles (S2 Fig). The assay showed good parallelism (S3 Fig).

### Midkine Sandwich ELISA Assay

MDK sandwich ELISA was performed as previously described using a commercial kit (Biovendor, Czech Republic) with modifications [12]. Intra-assay CV was 3.4% at high concentration (0.7 ng/mL) and 5.2% at low concentration (0.25 ng/mL). Inter-assay CV was 12.3% at low concentration. The limit of detection was 0.009 ng/mL. There was no cross-reactivity with up to 50 ng/mL of PTN (S1B Fig).

### Thyroglobulin Assay

50 μL of buffer containing thyroid tissue from an FNA needle was diluted 10-fold in normal saline and the concentration of Tg was measured with a chemiluminescent immunometric assay (Immulite 2000XPi, Siemens, UK) according to the manufacturer’s instruction and as previously described [12].

### BRAF mutation analysis

DNA was extracted on a Qiacube semiautomated robotic device (Qiagen, Valencia, CA) using either the QIAamp DNA Mini Kit (Qiagen) from 17 FNA washout samples and 6 frozen tissue samples, or the QIAamp DNA FFPE Tissue Kit (Qiagen) for paraffin-embedded tissue sections, according to the instructions of the manufacturer. *BRAF* T1799A (V600E) mutational analysis was performed using the PrimePCR ddPCR mutation detection assay (BIO-RAD, Hercules, CA) on a BIO-RAD QX200 droplet digital PCR (ddPCR) system. Each reaction included 10 μl of 2x ddPCR supermix for probes (no dUTP), 1 μl of *BRAF* V600E primer/probe mix (FAM), 1 μl of BRAF wild type primer/probe mix (HEX), and 40–100 ng of genomic DNA. The presence of mutation and the fractional abundance of the mutant allele was determined with QuantaSoft v.1.7 (BIO-RAD).

### Pleiotrophin Immunohistochemistry

Tissues were formalin-fixed, embedded in paraffin, and cut into 5-μm-thick sections which were deparaffinized and rehydrated in graded alcohol. For antigen retrieval, sections were placed in citrate buffer at 120°C using a pressuer cooker for 10 minutes. After blocking with 1.5% normal rabbit serum in Tris-buffered saline and Tween 20 (TBST) for 60 minutes at room temperature, the slides were incubated with a goat polyclonal antibody raised against human PTN (Cat# AF-252-PB, R&D Systems, Minneapolis, MN) at 1:250 dilution in 1.5% serum in TBST overnight at 4°C and then visualized with a biotinylated anti-goat IgG secondary antibody (1:200) using the Vectastain ABC (Goat IgG, PK-6105) and DAB kits (Vector Laboratories, Inc. Burlingame, CA) and counterstained with haematoxylin. Omission of the primary antiserum was used as a negative control and mouse embryo slides were used as a positive control (S5 Fig). Slides were scanned using a ScanScope XT digital slide scanner and viewed using ImageScope software (Aperio Technologies, Inc., Vista, CA).

### Statistical analysis

All FNA samples from each nodule were averaged to obtain a single mean value. After log transformation, the PTN concentration, Tg concentration and PTN/Tg ratio were compared between histological groups using t-tests and ANOVA with post-hoc Bonferroni correction for multiple comparison. The relationship between PTN and Tg was evaluated after log transformation by general linear model with nodule number as a covariate. The relationship between PTN/Tg and MDK/Tg was evaluated by Pearson regression after log transformation. Statistical analysis was performed using SPSS, version 12 (IBM, NY).

## Results

### Characteristics of subjects and nodules

A total of 103 nodules from 74 subjects (age 47 ± 12 y, 15 males) were studied by *ex vivo* FNA at the time of procurement, immediately after thyroidectomy. Histological examination revealed 23 nodules with PTC (10 classic, 6 tall cell, 6 follicular variant, and 1 undetermined), 1 nodule with medullary thyroid cancer and 79 benign nodules (72 adenomatoid nodules, 4 follicular adenomas and 3 hyperplastic nodules). Characteristics of nodules with PTC are shown in Table 1.

**Table 1:** Characteristics of papillary thyroid cancers (PTCs) studied.

Diagnosis	Tumor size	Metastasis^a^	MDK/Tg(ng/mg)	PTN/Tg (ng/mg)	BRAF mutation
PTC, classic					
	2.4 cm	5/6 LN	57.7	2.7	+
	0.5 cm		11.8	5.7	-
	0.9 cm		64.1	89.1	+
	3.0 cm		169.9	85.5	+
	1.0 cm		2.4	0.6	-
	4.7 cm	1/1 LN	730.0	54.0	+
	1.0 cm		3.6	4.6	+
	0.7 cm		16.7	0.5	+
	1.8 cm	1/2 LN	NA	0.1	+
	1.6 cm		37.0	1.8	+
PTC, tall cell variant					
	5.0 cm	4/33 LN	262.0	10.7	+
	1.8 cm	1/1 LN	22.4	0.5	+^c^
	1.5 cm	1/1 LN	0.5	0.4	+^c^
	1.5 cm	1/1 LN	0.3	12.4	+^c^
	3.0 cm	2/7 LN	606.0	19.2	+
	2.6 cm		646.0	59.0	+
PTC, follicular variant					
	0.8 cm		1.5	1.0	+
	1.0 cm		0.6	1.3	-^d^
	0.5 cm		1.7	4.0	-
	0.5 cm		4.5	0.3	-
	1.8 cm	1/2 LN	0.2	0.5	-
	1.1 cm		0.9	0.4	-^d^
PTC, undetermined^b^					
	5.0 cm		103.0	2.9	+

^a^LN lymph nodes (number of positive lymph nodes/ total lymph nodes examined).

^b^Histologically, inconclusive but clinically and radiologically malignant

^c^Different nodules from a subject

^d^Different nodules from a subject

### Association of PTN and Tg concentrations in FNA samples

PTN concentrations were positively associated with Tg concentrations in FNA washout samples from benign nodules (analysis included all individual passes, R^2^ = 0.04, *P* < 0.001, S4 Fig). Since this correlation likely occurred because both PTN and Tg concentrations in the washout fluid were dependent on the amount of thyroid tissue present in the sample, PTN levels were normalized to Tg levels as PTN/Tg, ng/mg, to correct for the amount of thyroid tissue.

### PTN concentrations and PTN/Tg ratio in ex vivo FNA samples

**Fig 1:**
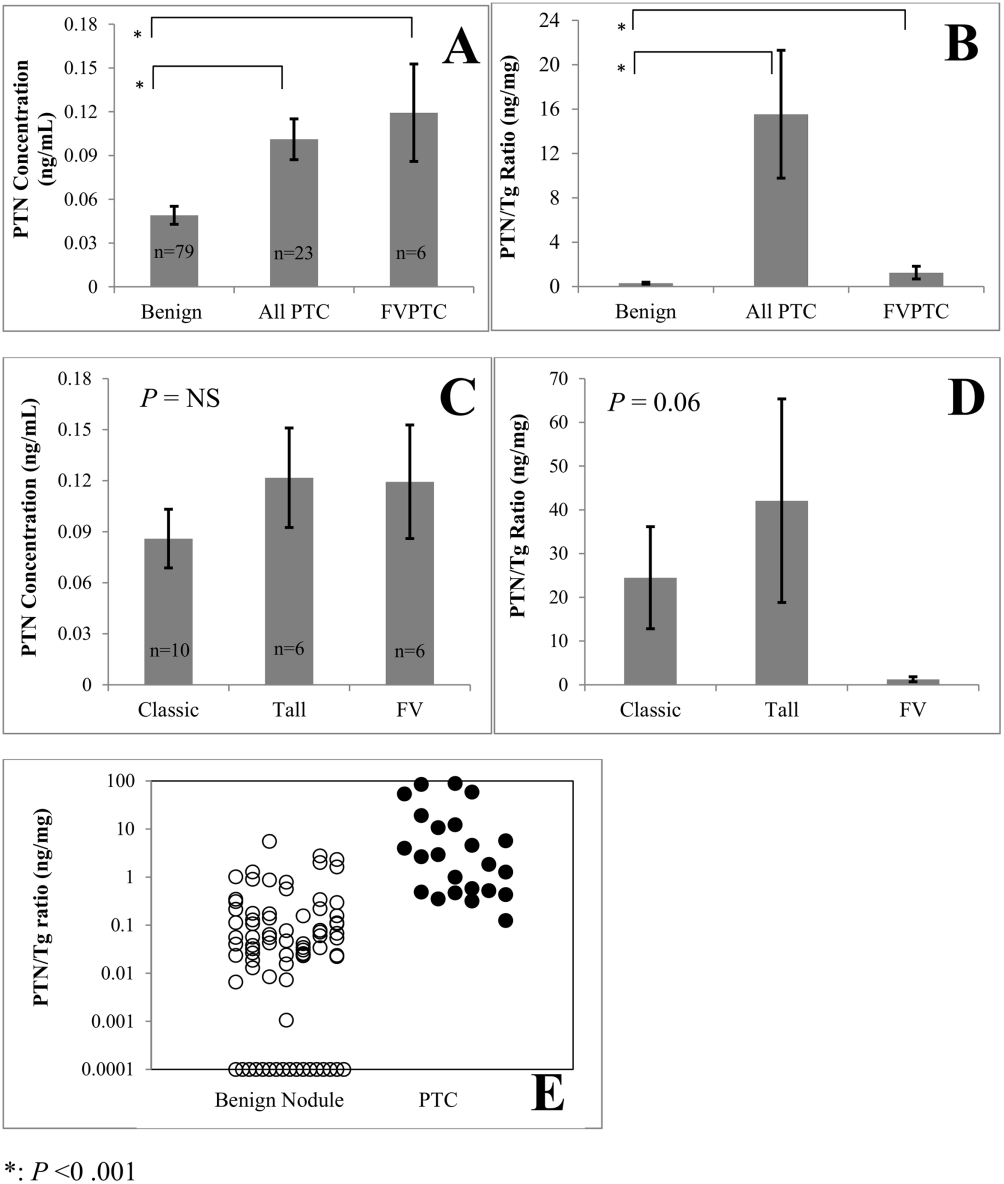
Pleiotrophin (PTN) concentrations and pleiotrophin/thyroglobulin ratios (PTN/Tg) in benign nodules and papillary thyroid cancer (PTC). Samples were obtained by *ex vivo* fine needle aspiration; PTN and Tg were measured by immunoassay. PTN concentrations (mean ± SEM) were higher in PTC (including all subtypes) and in the subset of follicular variant PTC (FVPTC) than in benign nodules (A). Similarly, PTN/Tg was greater in PTC (including all subtypes) and in the subset of FVPTC than in benign nodules (B). PTN concentrations did not differ significantly among classic, tall cell variant, and FVPTC (C). PTN/Tg tended (*P* = NS) to be lower in FVPTC than in other subtypes (D). Scatterplot showing PTN/Tg values of all nodules. Closed symbols, PTC; open symbols, benign nodules (E). Values less than 0.001 are displayed as equal to 0.001.

PTN concentrations in PTC were significantly higher than in benign nodules (0.1 ± 0.01 vs 0.05 ± 0.01 ng/mL, mean ± SEM, p<0.001). PTN concentrations in the subset of follicular variant papillary thyroid cancer (FVPTC) were also higher than in benign nodules (0.12 ± 0.03 vs 0.05 ± 0.01 ng/mL, p<0.001, Fig 1A).

Tg concentrations were lower in PTC than in benign nodules (112 ± 30 vs 926 ± 88 ug/mL, *P* < 0.001). In the FVPTC subgroup, Tg concentrations also tended to be lower than in benign nodules but the difference was not statistically significant (203 ± 80 vs 926 ± 88 ug/mL ng/mL, *P* = NS). The ratio of PTN to Tg (ng/mg) was higher in PTC than in benign nodules (16 ± 6 vs 0.3 ± 0.1 ng/mg, *P* < 0.001, Fig 1B and 1E), and also significantly higher in FVPTC than in benign nodules (1.3 ± 0.6 vs 0.3 ± 0.1 vs ng/mg, *P* < 0.001)(Fig 1B). PTN/Tg was also elevated in the one nodule containing medullary thyroid cancer (2.9 ng/mg).

Among PTCs, there was no difference in PTN concentrations among classic, tall cell variant and FVPTCs (0.09 ± 0.02, 0.12 ± 0.03 and 0.12 ± 0.03 ng/mL, respectively, *P* = NS)(Fig 1C). However, FVPTC had the lowest the PTN/Tg ratios (24.5 ± 11.7, 42.1 ± 23.3 and 1.3 ± 0.6 ng/mg, respectively, *P* = 0.06)(Fig 1D).

Of 10 benign nodules with the highest PTN/Tg ratio, 2 occurred in patients with Graves’ disease (2 of 2 subjects with Graves’ disease), 1 in a subject with a follicular adenoma (1 of 4 subjects with follicular adenoma), 1 in a patient with chronic lymphocytic thyroiditis (1 of 3 subjects with chronic lymphocytic thyroiditis), 1 in a benign nodule adjacent to PTC and 5 in benign nodules without other significant histological findings.

The PTN/Tg ratio had no association with nodule size or the presence of lymph node metastasis (data not shown).

### Association between the PTN/Tg and MDK/Tg and bivariate analysis

**Fig 2:**
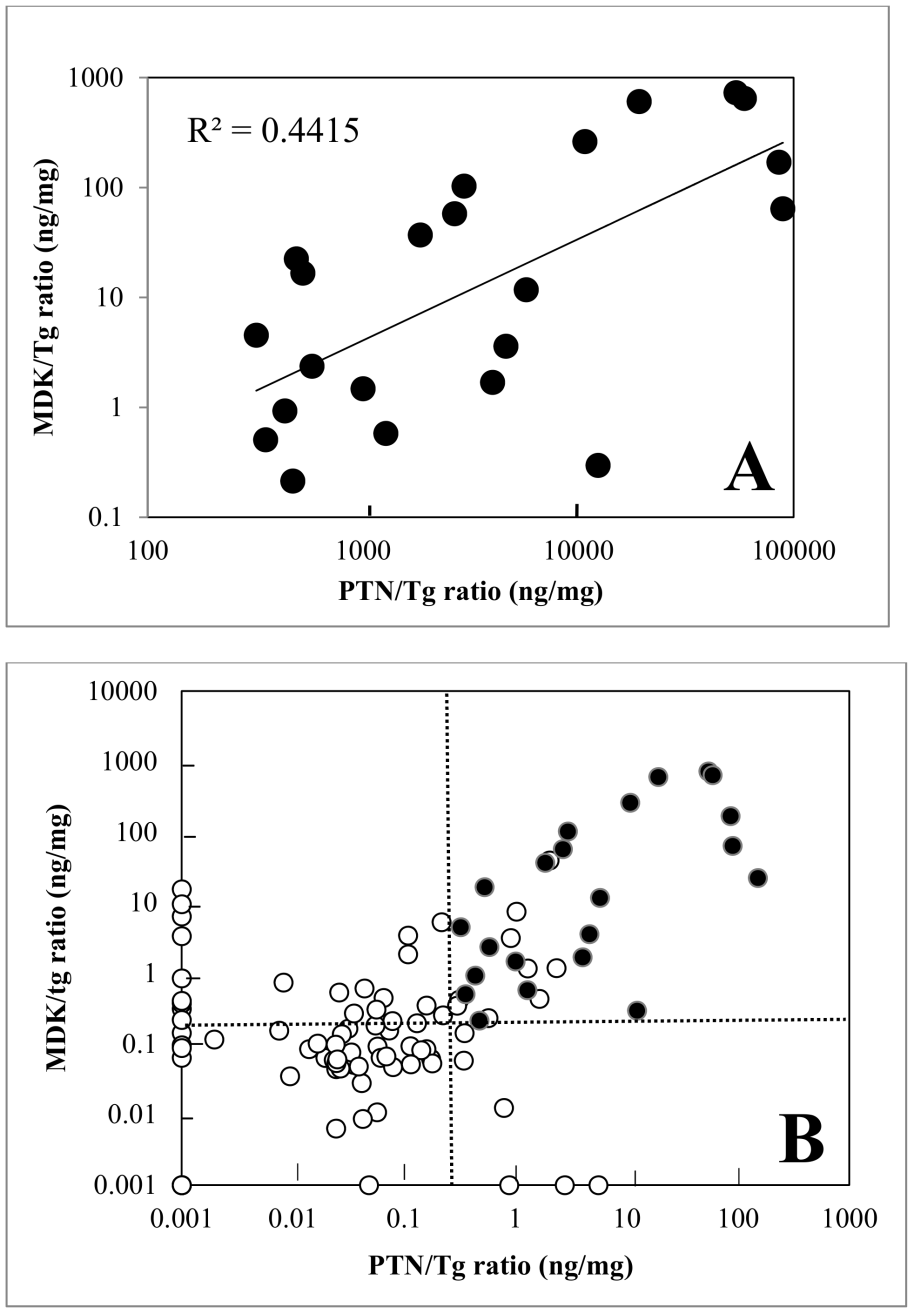
**Association between PTN/Tg and MDK/Tg ratios among all PTC nodules studied (A)**. PTN/Tg and MDK/Tg were positively correlated (R^2^ = 0.44, *P* = 0.001). **Bivariate analysis of PTN/Tg and MDK/Tg ratios (B)**. All PTCs had MDK/Tg greater than 0.2 ng/mL (horizontal dashed line) and PTN/Tg greater than 0.13 ng/mL (vertical dashed line). Values less than 0.001 are displayed as equal to 0.001. Closed circles, PTC; open circles, benign nodules.

Both MDK/Tg and PTN/Tg were measured in 22 PTCs and 77 benign nodules. There was no association between the PTN/Tg and MDK/Tg among benign nodules. However, there was a positive correlation between MDK/Tg and PTN/Tg among PTCs (R^2^ = 0.44, *P* = 0.001) (Fig 2A).

A bivariate plot of all nodules with both PTN/Tg and MDK/Tg measured revealed that all PTCs had MDK/Tg greater than 0.2 ng/mL and PTN/Tg greater than 0. 13 ng/mL (Fig 2B). Of the 35 nodules that met both these criteria, 23 were malignant (100% of PTC) and 12 were benign (15% of benign nodules), yielding a sensitivity of 100% and a specificity of 85%.

### Association between PTN/Tg ratio and BRAF mutation

Among 23 PTC nodules, 16 had the *BRAF* V600E mutation. The PTN/Tg ratio tended to be higher in *BRAF*-positive than in *BRAF*-negative nodules but the difference did not reach statistical significance (21.5 ± 7.9 vs 1.8 ± 0.8, *P* = 0.095).

### Confirmation of PTN expression using immunohistochemistry

**Fig 3:**
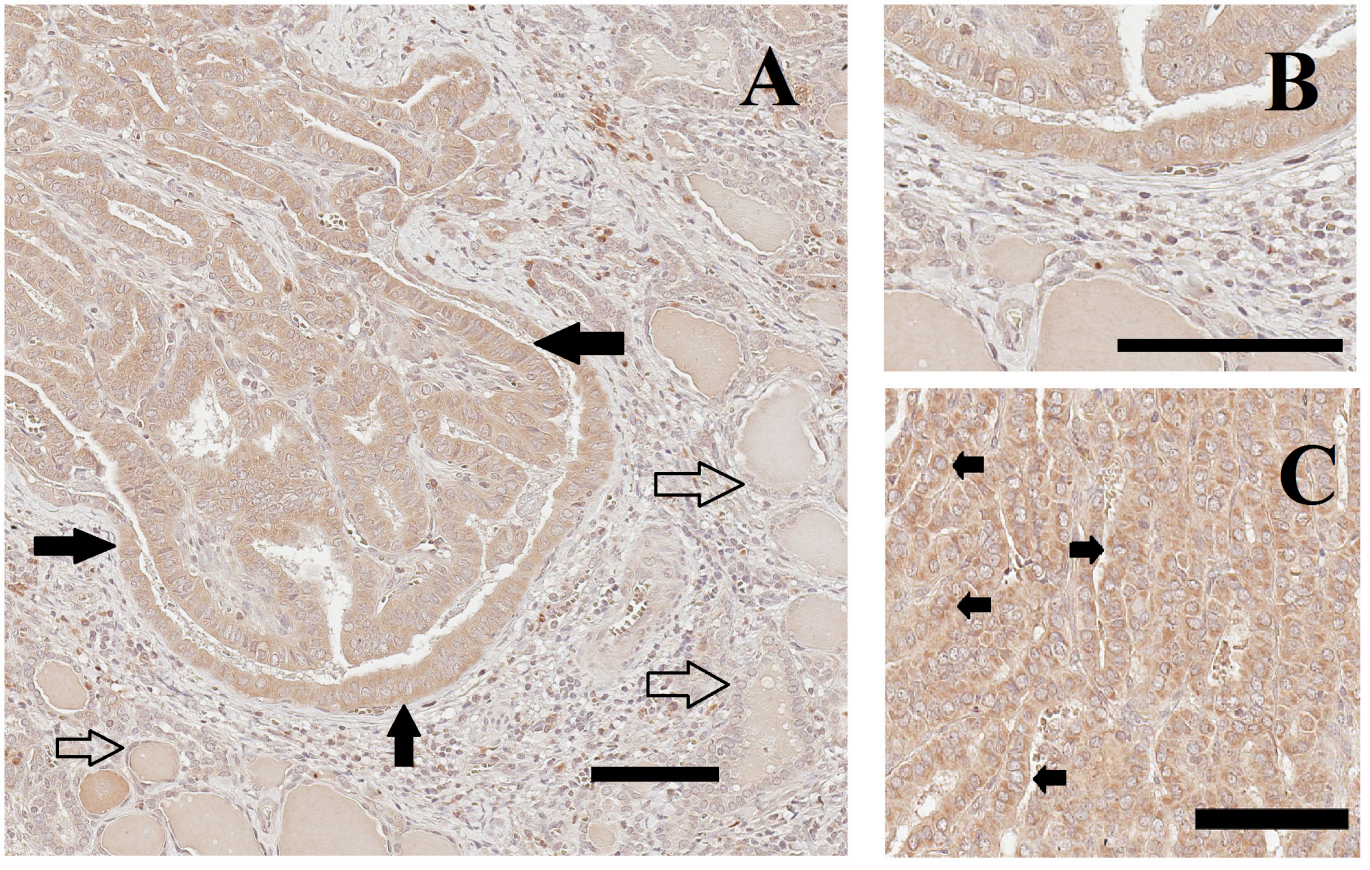
Immunohistochemical staining for PTN. A) Histological sections containing tall cell variant PTC were immunostained for PTN (brown color) and counterstained with hematolxylin (purple color). Immunohistochemical staining was more intense in the neoplastic thyroid epithelial cells within the PTCs (closed arrows) than in in nearby normal thyroid epithelial cells (open arrows). Some stromal cells in the adjacent connective tissue also showed immunohistochemical staining. B) Higher magnification of PTC and normal tissue from the same section as in panel A. C) Immunohistochemical straining of classic PTC that shows perinuclear location of PTN (arrows). Size bar, 100 μm.

Immunohistochemical staining of tissue sections revealed that PTN immunostaining was more intense in those neoplastic thyroid epithelial cells within the PTCs than in the nearby normal thyroid epithelial cells. (Fig 3A–3C). Some scattered stromal cells in the adjacent connective tissue also showed immunohistochemical staining. Within thyroid epithelial cells, the PTN staining was primarily cytoplasmic and perinuclear (Fig 3C).

## Discussion

We found that PTN was measurable in FNA samples obtained *ex vivo* from thyroidectomy specimens and that the PTN concentrations were higher in PTC than in benign nodules. Similarly, the PTN/Tg ratio was greater in PTC than in benign nodules. We initially chose to use Tg as a measure of tissue content. However, we found that the level of Tg in samples from PTC was lower than in samples from benign nodules, suggesting that Tg expression might be lower in malignant cells, consistent with a prior study [14], and therefore Tg may not simply be a measure of tissue content. However, from a pragmatic standpoint, this effect is fortuitous because normalization of PTN to Tg enhanced the separation between malignant and benign samples.

PTN expression by PTCs was confirmed by immunohistochemistry. The immunohistochemical findings suggest that PTN is overexpressed by the neoplastic thyroid epithelial cells themselves, primarily in a perinuclear and cytoplasmic localization as reported in other tissues [15–16]. However, staining for PTN was also observed in the adjacent stromal cells raising the possibility that other cells might also contribute to the elevated PTN measured by ELISA in PTC.

The finding that PTN is elevated in PTC suggests that PTN overexpression may promote growth of PTCs, which has also been suggested for other cancers, such as ovarian [17], pancreatic [18], glioblastoma [19], prostate cancer [20] and breast cancer [21]. For example, in a breast cancer model, PTN overexpression stimulated remodeling of the microenvironment, tumor angiogenesis, and rapid tumor growth [22]. However, in our study, we did not observe an association between the PTN/Tg ratio and the size of the nodules or the presence of lymph node metastasis. We also did not find a significant association between the PTN/Tg ratio and the presence of the BRAF V600E mutation. However, the sample size of our study is insufficient to definitively exclude associations with disease aggressiveness or genetic etiology.

We previously found that the concentration of MDK, a heparin-binding growth factor related to PTN, was higher in PTC than in benign nodules [12]. In the current study, we found that MDK/Tg and PTN/Tg were positively correlated in PTCs. However, one important difference is that, in FVPTC, the PTN/Tg was elevated whereas the MDK/Tg showed values overlapping those of benign nodules. This finding is of particular interest because FVPTC is often difficult to distinguish from benign follicular lesions by cytology, often requiring histological evaluation [7].

A bivariate plot of PTN/Tg and MDK/Tg showed strong clustering of PTC samples, such that all malignancies had MDK/Tg greater than 0.2 ng/mg and PTN/Tg greater than 0.13 ng/mg. Only 15% of benign nodules satisfied both these criteria (Fig 2B). The observation that the PTN/Tg ratio and the MDK/Tg ratio are elevated in PTC compared to benign nodules raises the possibility that measurement of PTN, MDK, and Tg in thyroid FNA samples might provide useful adjunctive diagnostic information to cytologic examination, as has been demonstrated by RNA profiling [23], mutational analysis [24] and other molecular approaches [25]. However, to establish a clinically useful approach, there are important challenges that would need to be overcome, many of which reflect limitations in the current study. First, to measure PTN in FNA washout, sufficient thyroid tissue must be present. Thus, a dedicated FNA pass, separate from those required for cytology, may be required to obtain sufficient tissue as is being done with the commercially available gene expression classifier test [23]. Alternative possible approaches include developing a more sensitive PTN assay or washing the expelled needle with a smaller volume of buffer and performing the assay without dilution. Second, the current study was performed using FNA samples obtained *ex vivo* after thyroidectomy. Whether similar data would be observed with *in vivo*, percutaneous FNA sampling is unknown. We did not address this question because of the unavailability of dedicated *in vivo* FNA samples in this research study. Third, our approach may not be useful in patients with Graves’ disease or chronic lymphocytic thyroiditis; we observed elevated PTN/Tg ratios in benign nodules within thyroid glands affected by these autoimmune disease. Fourth, adaptation of these findings into an adjunctive clinical diagnostic test would require a substantially larger study to determine the sensitivity and specificity in subjects with indeterminant cytology. The current pilot study demonstrates a novel observation of elevated PTN/Tg in all types of PTC, but was not designed to rigorously validate a diagnostic test. Our study is designed for a proof-of-concept and used *ex vivo* FNA materials. Therefore, ROC analysis is not performed.

## Conclusions

In conclusion, our findings indicate that PTN concentrations and the PTN/Tg ratio in *ex vivo* FNA samples distinguish PTC from benign lesions, raising the possibility that this strategy may have adjunctive diagnostic utility to supplement cytology and other existing molecular methods. However, additional larger studies would be needed to validate this approach.

## Supporting Information

S1 Fig **Supplemental Figure 1A**. PTN cross-reactivity with MDK. **Supplemental Figure 1B**. MDK cross-reactivity with PTN.
(DOCX)

S2 Fig **Supplemental Figure 2A**. Stability in glass vs plastic tube. **Supplemental Figure 2B**. Stability at room temperature and during freeze and thaw cycle.
(DOCX)

S3 Fig Supplemental Figure 3. Parallelism of the PTN ELISA between the standard curve and serially diluted washout samples.
(DOCX)

S4 Fig Supplemental Figure 4. PTN concentrations were positively associated with Tg concentrations in FNA washout samples from benign nodules (analysis included all individual passes, R^2^ = 0.04, P < 0.001).
(DOCX)

S5 Fig Supplemental Figure 5. Positive and negative IHC control.
(DOCX)

S1 Method Supplemental Method. Pleiotrophin Sandwich ELISA Assay.
(DOCX)
