# Supplementary material for: Increased Pleiotrophin Concentrations in Papillary Thyroid Cancer
Source: PLoS One. 2016 Feb 25;11(2):e0149383. doi: 10.1371/journal.pone.0149383 (PMC4767803; doi:10.1371/journal.pone.0149383)
Supplement: S1 Fig — Supplemental Figure 1A. PTN cross-reactivity with MDK. Supplemental Figure 1B. MDK cross-reactivity with PTN. (DOCX) [file pone.0149383.s001.docx]

**S1 Fig.**

**Supplemental Fig 1A. PTN cross-reactivity with MDK.** PTN concentrations were measured in 1) the buffer without PTN, 2) the buffer + 50 ng/mL of human recombinant MDK, 3) the buffer spiked with 1.2 ng/mL of human recombinant PTN, 4) the buffer spiked with 1.2 ng/mL human recombinant PTN and 50 ng/mL of MDK. There was no cross-reactivity with 50 ng/mL of MDK.

**Supplemental Fig 1B. MDK cross-reactivity with PTN**. MDK concentrations were measured in 1) the buffer without MDK, 2) the buffer + 50 ng/mL of human recombinant PTN, 3) the buffer spiked with 1.2 ng/mL of human recombinant MDK, 4) the buffer spiked with 1.2 ng/mL human recombinant MDK and 50 ng/mL of MDK. There was no cross-reactivity with 50 ng/mL of PTN.
