# Supplementary material for: Increased Pleiotrophin Concentrations in Papillary Thyroid Cancer
Source: PLoS One. 2016 Feb 25;11(2):e0149383. doi: 10.1371/journal.pone.0149383 (PMC4767803; doi:10.1371/journal.pone.0149383)
Supplement: S2 Fig — Supplemental Figure 2A. Stability in glass vs plastic tube. Supplemental Figure 2B. Stability at room temperature and during freeze and thaw cycle. (DOCX) [file pone.0149383.s002.docx]

**S2 Fig.**

**Supplemental Figure 2A. Stability in glass vs plastic tube**. Buffer was spiked with PTN at concentration 1.2 ng/mL and placed in glass and plastic tubes for 1 and 2 hours at room temperature. Then, PTN concentrations were measured. The finding shows that glass adsorb PTN.

**Supplemental Figure 2B. Stability at room temperature and during freeze and thaw cycle.** Buffer was spiked with PTN at concentration 1.2 ng/mL and placed at room temperature for 2 hours. The spiked buffer was frozen and thawed up to 3 times over a month period of time. Then, PTN concentrations were measured. The result showed stable PTN concentrations. RT: room temperature. FT: freeze-thaw cycle.
