# Supplementary material for: Increased Pleiotrophin Concentrations in Papillary Thyroid Cancer
Source: PLoS One. 2016 Feb 25;11(2):e0149383. doi: 10.1371/journal.pone.0149383 (PMC4767803; doi:10.1371/journal.pone.0149383)
Supplement: S3 Fig — Parallelism of the PTN ELISA between the standard curve and serially diluted washout samples. (DOCX) [file pone.0149383.s003.docx]

**Supplemental Figure 3. Parallelism of the PTN ELISA between the standard curve and serially diluted washout samples**. An FNA washout sample from a benign nodule was spiked with 0.1 ng/mL of recombinant human PTN and was diluted 2, 3, 5 and 10 fold into assay buffer (PBSTA) and then assayed for PTN. Black bars, measured PTN concentration; Gray bars, measured value multiplied by the dilution factor.
