# Supplementary material for: Increased Pleiotrophin Concentrations in Papillary Thyroid Cancer
Source: PLoS One. 2016 Feb 25;11(2):e0149383. doi: 10.1371/journal.pone.0149383 (PMC4767803; doi:10.1371/journal.pone.0149383)
Supplement: S4 Fig — PTN concentrations were positively associated with Tg concentrations in FNA washout samples from benign nodules (analysis included all individual passes, R2 = 0.04, P < 0.001). (DOCX) [file pone.0149383.s004.docx]

**S4 Fig**

**Supplemental Figure 4.** PTN concentrations were positively associated with Tg concentrations in FNA washout samples from benign nodules (analysis included all individual passes, R^2^ = 0.04, P < 0.001)
