# Supplementary material for: Increased Pleiotrophin Concentrations in Papillary Thyroid Cancer
Source: PLoS One. 2016 Feb 25;11(2):e0149383. doi: 10.1371/journal.pone.0149383 (PMC4767803; doi:10.1371/journal.pone.0149383)

**S5 Fig.**

**Supplemental Figure 5. Positive and negative IHC control.** Mouse embryo was used as a positive control because multiple embryonic tissues express PTN (left section). Omission of the primary antiserum was used as a negative control (right section).


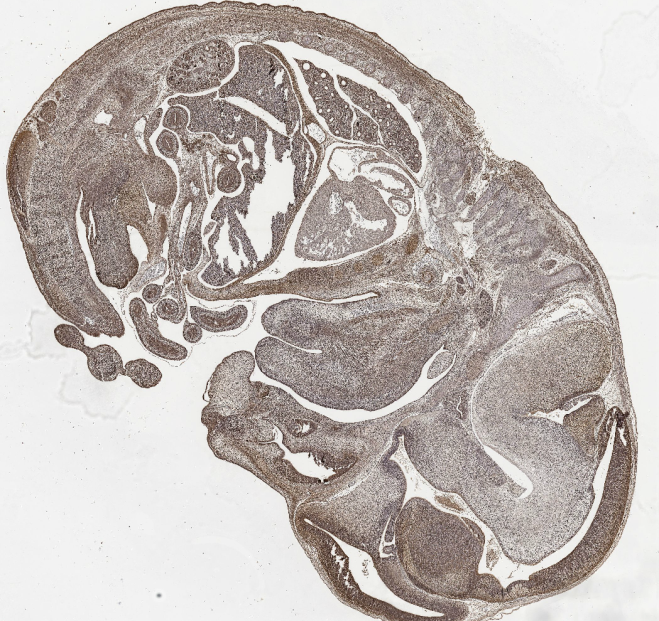

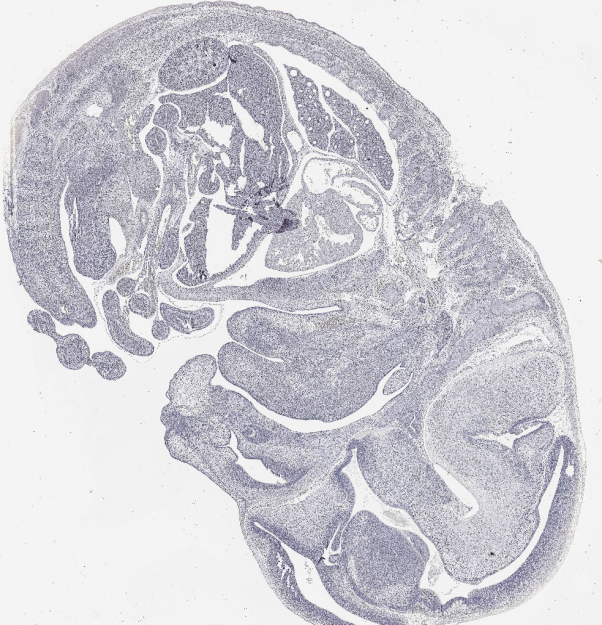

Supplement: S5 Fig — Positive and negative IHC control. (DOCX) [file pone.0149383.s005.docx]
