# Supplementary material for: Increased Pleiotrophin Concentrations in Papillary Thyroid Cancer
Source: PLoS One. 2016 Feb 25;11(2):e0149383. doi: 10.1371/journal.pone.0149383 (PMC4767803; doi:10.1371/journal.pone.0149383)
Supplement: S1 Method — Pleiotrophin Sandwich ELISA Assay. (DOCX) [file pone.0149383.s006.docx]

***S1. Supplemental Method***

*Pleiotrophin Sandwich ELISA Assay*

Pleiotrophin sandwich ELISA was developed in our lab. The mouse anti-pleiotrophin monoclonal antibody (3B10) was produced at Dr. Anton Wellstein’s laboratory. The antibody was diluted to 0.5 ug/mL in PBS and 100 uL/well was incubated in a 96 well plate at 4°C overnight. The wells were washed 3 times with 250 uL per well PBST (PBS, 0.05% Tween20). The wells were blocked with 250 uL per well of PBS containing 3% BSA and 0.2% tween 20 for 2 hours at 4°C. Without washing, the plate was inverted and dried by tapping vigorously against a paper towel. 100 uL of buffer containing thyroid tissue from an FNA needle was diluted in 200 uL of PBSTA (PBS, 1% BSA, 0.5% Tween 20). 100 uL of the diluted samples was pipetted in duplicate into 96-well plates. The plate was incubated with gentle agitation at room temperature for 2 hours and then washed 3 times with 0.25 ml per well of PBST. After tapping the inverted plate to remove residual fluid, a biotinylated anti-human pleiotrophin goat IgG was added at a concentration of 500 ng/mL in a 0.9 % normal saline containing 5.7 meq/L calcium chloride, 0.5% BSA at pH 6. The plate was incubated with gentle agitation at room temperature for an hour. Then, the wells were washed 5 times with 0.25 ml of PBST per well. After tapping, 100 ul of streptavidin-HRP conjugate solution was added at a concentration of 50 ng/mL in PBS to each well and was incubated at room temperature for 30 minutes with gentle agitation. After washing 5 times with PBST and tapping, 100 uL of TMB was added to each well. The plate was covered with aluminum foil and incubated for 7 minutes at room temperature. Color development was stopped by adding 100 μl of stop solution. The absorbance of each well was measured using a microplate reader set to 450 nm.
